# Assessing the functional potential of conditioned media derived from amniotic epithelial stem cells engineered on 3D biomimetic scaffolds: An in vitro model for tendon regeneration

**DOI:** 10.1016/j.mtbio.2024.101001

**Published:** 2024-02-18

**Authors:** Valentina Russo, Giuseppe Prencipe, Annunziata Mauro, Mohammad El Khatib, Arlette A. Haidar-Montes, Nico Cambise, Maura Turriani, Johannes Stöckl, Peter Steinberger, Loreto Lancia, Matthias Schnabelrauch, Paolo Berardinelli, Barbara Barboni

**Affiliations:** a Unit of Basic and Applied Sciences, Faculty of Biosciences and Agro-Food and Environmental Technologies, University of Teramo, 64100 Teramo, Italy; b Research & Development Department, Assut Europe S.p.A., Magliano dei Marsi, 67062 L'Aquila, Italy; c Centre for Pathophysiology, Infectiology and Immunology, Institute of Immunology, Medical University of Vienna, 1090 Vienna, Austria; d Department of Biotechnological and Applied Clinical Sciences, University of L'Aquila, 67100 L'Aquila, Italy; e Department of Biomaterials, INNOVENT e. V., 07745 Jena, Germany

**Keywords:** Tenogenic differentiation, Paracrine effect, 3D electrospun PLGA scaffolds, Amniotic epithelial stem cells (AECs), Immunomodulation

## Abstract

Tendon diseases pose a significant challenge in regenerative medicine due to the limited healing capacity of this tissue. Successful tendon regeneration requires a combination of angiogenesis, immune response, and tenogenesis processes. An effective tendon engineering (TE) strategy must finely tune this systems’ interplay toward homeostasis.

This study explores *in vitro* the paracrine influence of amniotic epithelial stem cells (AECs) engineered on a validated 3D electrospun PLGA scaffolds on HUVECs (angiogenesis), PBMCs/Jurkat (immune response), and AECs (tenogenic stem cell activation).

The results revealed the role of scaffold's topology and topography in significantly modulating the paracrine profile of the cells. In detail, AECs basal release of bioactive molecules was boosted in the cells engineered on 3D scaffolds, in particular VEGF-D, b-FGF, RANTES, and PDGF-BB (*p* < 0.0001 vs. CM_CTR_). Moreover, biological tests demonstrated 3D scaffolds' proactive role in potentiating AECs' paracrine inhibition on PBMCs proliferation (CM_3D_*vs.* CTR, *p* < 0.001) and LPS-mediated Jurkat activation with respect to controls (CM_3D_ and CM_2D_*vs.* CTR, *p* < 0.01 and *p* < 0.05, respectively), without exerting any *in vitro* pro-angiogenic role in promoting HUVECs proliferation and tubule formation. Teno-inductive paracrine ability of AECs engineered on 3D scaffolds was assessed on co-cultured ones, which formed tendon-like structures. These latter demonstrated an upregulation of tendon-related genes (*SCX, THBS4, COL1,* and *TNMD*) and the expression TNMD and COL1 proteins.

Overall, this research underscores the pivotal role of the 3D topology and topography of PLGA tendon mimetic scaffolds in orchestrating effective tendon regeneration through modulating cell behavior and crosstalk between engineered stem cells and different subpopulations in the damaged tendon.

## Highlights

• Tendon biomimetic PLGA 3D scaffolds' topology fine-tunes amniotic epithelial stem cells (AECs) paracrine function.
• AECs engineered on 3D scaffolds release angiogenic, immunomodulatory and teno-inductive molecules.
• 3D scaffolds as an *in vitro* model to predict regenerative crosstalk between stem cells and damaged tendon subpopulations.

## Graphical abstract

**Figure:**
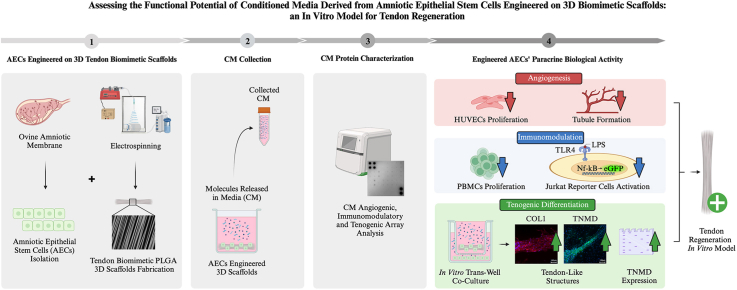

## 1 Introduction

Tendons are dense connective tissues that connect muscles to bones, and when injured, they have limited regenerative capacity [[1], [2], [3]]. Traditional treatments for tendon injuries, such as pharmacological symptomatic treatments, (i.e. non-steroidal anti-inflammatory drugs, and corticosteroids), and surgical repair (i.e. autografts, allografts, and xenografts) [1,2,[4], [5], [6], [7]], are frequently suboptimal since they provide a limited functional recovery, exposing the repaired tissue to a high risk of re-injury.

The healing process of an injured tendon usually includes three sequential and overlapping phases: inflammation, proliferation and remodeling [8]. The appropriate time of tissue engineering (TE) application is during the inflammatory phase that must support the anatomical asset of the tissue and at the same time must also avoid an excessive and persistent inflammation, which is responsible for an impaired tendon regeneration [8]. In this phase, an active angiogenesis is required for a rapid and transitory formation of the intravascular plexus, with a Vascular Endothelial Growth Factor (VEGF) overexpression [9,10], essential for providing oxygen, nutrients, and immune cells to support tendon regeneration. However, the formed vascular plexus over time must undergo to a specific remodeling and a consequent VEGF downregulation for a correct recovery of tendon microarchitecture [9,11,12].

Contextually, the immune system that operates both during the inflammatory and the proliferation phases, plays a dual role in tendon regeneration. In the initial stages of injury, immune cells are involved in the inflammatory phase and are recruited to the site of injury [3]. Various substances, including cytokines and growth factors, are released from immune cells to help regulating the healing process by creating a favorable environment for tissue repair. The immune system, along with tenocytes, also contribute to the production and remodeling of the extracellular matrix (ECM), crucial to restore tendon's functionality [3,12,13].

However, uncontrolled inflammation and an imbalance in the immune response may hinder regeneration, leading to scar tissue formation instead of a healthy tendon tissue [3,14]. All these crucial aspects have to be considered and controlled also when implanting a scaffold *in vivo* in case of tendon ruptures. Indeed, an implanted scaffold can either determine the progress in tissue regeneration avoiding the risk of inducing an aberrant inflammatory response or the failure of the implanted scaffold provoking a foreign body response (FBR) within the host tissue [15]. The *in situ* immune response must be regulated by modulating the interplay amongst the immune system, blood vessels, and somatic/progenitor cells, which are involved in the ECM remodeling. The interplay of these three elements is fundamental for developing TE strategies to enhance tendon regeneration.

The scaffolds must be designed to mimic the structure of the ECM, providing at the same time a supportive microenvironment for different cells districts, enhancing their biological activity [16]. To harness scaffold's inductive potentialities, it is also crucial to identify the optimal stem cell source to engineer within, specifically one with recognized tendon regenerative capabilities. Amniotic epithelial stem cells (AECs) have proven to be highly effective in tendon regeneration due to their potent anti-inflammatory and pro-regenerative paracrine effects [9,12,13,[16], [17], [18], [19], [20], [21]]. Moreover, these cells exhibit a remarkable ability to undergo step-wise teno-transdifferentiation both *in vitro* [16,[18], [19], [20], [21], [22]], as well as *in vivo* [9,12,13,23]. AECs' teno-differentiation properties are further potentiated when they are engineered on the developed poly (lactide-*co*-glycolide) (PLGA) 3D tendon‐hierarchical scaffolds at both molecular and paracrine levels [16].

Recent advances in regenerative medicine underscore the pivotal role of extracellular vesicles, particularly exosomes and miRNAs, as key contributors to paracrine-mediated intercellular communication and tissue repair [[24], [25], [26]]. These discoveries highlight a new dimension in scaffold-based TE, emphasizing the need to explore the paracrine inductive effects of the scaffold, a critical aspect for predicting their safety and efficacy before moving towards pre- and clinical translation.

The interplay between materials’ topography and topology and their inductive paracrine role on stem cells intercepts a new field that aims to dynamically study the material properties that, especially for tendon TE, has not been systematically studied before [16,24,[27], [28], [29]]. Moreover, a deep analysis of long-term paracrine inductive effect of the scaffold is essential to predict their safety and efficacy before moving towards pre- and clinical translation.

Based on these premises, the present research aimed to determine whether a validated 3D PLGA scaffold, which mimics native tendon hierarchical architecture and mechanical properties [16], could address the engineered AECs to release paracrine molecules that may have a functional role in modulating angiogenesis, immune response, and tenogenesis. In order to verify the effect not only of the topography but also of the topology (three dimensionality) of the constructs, the characterized conditioned media (CM) derived from AECs-engineered fleeces (AECs-2D) and 3D scaffolds (AECs-3D) were tested *in vitro* for their biological impact on target cells of the three systems: human umbilical vein endothelial cells (HUVECs), two typologies of immune cells: Peripheral Blood Mononuclear Cells (PBMCs) and Jurkat reporter cells, and stem cells with teno-differentiative attitude (AECs) to reproduce in culture the process of blood vessel formation, immunomodulation and teno-differentiation, respectively.

## 2 Materials and methods

### 2.1 Fabrication and use of PLGA constructs

Poly (lactide-*co*-glycolide) (PLGA, PLG8523) fleeces (2D) and 3D scaffolds (3D) were fabricated as previously reported [16]. Briefly, PLGA fleeces were electrospun using a commercial E-Spintronic electrospinning apparatus (Erich Huber, Gerlinden, Germany), equipped with a cylindrical metallic drum rotating collector. The collected fleeces were cut into rectangular pieces (10 mm × 7 mm) and the strips’ widths were rolled manually to obtain 3D scaffolds (representative photographs of PLGA fleeces and 3D scaffolds are shown in Supplementary 1) [16].

Before being used for biological experiments, PLGA fleeces and 3D scaffolds were sterilized in 70% ethanol (EtOH) as detailed previously [16,[19], [20], [21]]. Afterwards, both constructs were conditioned for 1 h incubation at 38.5 °C with 5% CO_2_ in cell culture growth medium (GM) composed of α-MEM supplemented with 10% FBS, 1% ultraglutamine, 1% amphotericin, and 1% penicillin/streptomycin [16,[19], [20], [21]].

### 2.2 Ethic statement

AECs were isolated from the amniotic membranes of slaughtered Appenninica breed sheep, considered as waste reproductive tissues of animals processed for food consumption. Contextually, PBMCs were isolated from blood collected immediately after sheep slaughtering. Accordingly, no ethical statement is required.

### 2.3 Isolation and culture of ovine AECs

**Fig. 1:**
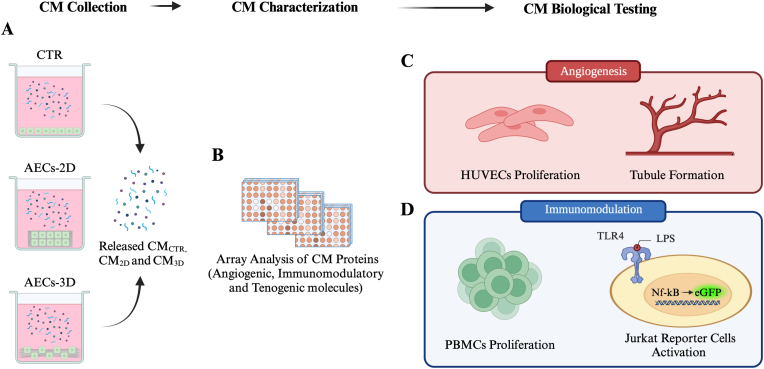
Experimental design for: (A) CM collection from CTR (CM_CTR_) and from AECs-2D (CM_2D_) and AECs-3D (CM_3D_); (B) CMs content evaluation by membrane antibody array; CMs biologically tested in terms of (C) angiogenesis on HUVECs, and of (D) immunomodulation on PBMCs and Jurkat reporter cells.

Ovine AECs were collected from the epithelial layer of the amniotic membrane at mid-pregnancy, and subsequently counted and characterized as previously reported [9,13,16,22,30]. Briefly, AECs were assessed for their negativity to hemopoietic markers (CD14, CD58, CD31, and CD45), positivity to both surface adhesion molecules (CD29, CD49f, and CD166) and stemness markers (TERT, SOX2, OCT4, and NANOG), low expression for MHC class I molecules, and the absence of MHC class II (HLA-DR) antigens [9,16,17,22,30,31]. The used OCT4 antibody mainly recognizes the pluripotency isoform OCT4A and in a less extent the OCT4B (ab18976, Abcam, Cambridge, United Kingdom). Moreover, freshly isolated AECs were confirmed to be negative to tendon-related gene markers: scleraxis (SCX), type I collagen (COL1), and tenomodulin (TNMD) [9,16,[19], [20], [21]]. Freshly isolated AECs were cultured in GM on Petri dishes (CTR) or seeded on PLGA fleeces (AECs-2D) and 3D scaffolds (AECs-3D) at the concentration of 0.05 × 10^6^ cells for 48 h at 38.5 °C with 5% CO_2_ (Fig. 1A) [16].

### 2.4 Conditioned media production

After 48 h of culture, all AECs conditions were starved for 24 h in serum-free GM and then the conditioned media (CMs) were collected as follow: AECs cultured on Petri dishes (CM_CTR_), on fleeces (CM_2D_) and on 3D scaffolds (CM_3D_) (Fig. 1A).

#### 2.4.1 Array analysis of AECs derived-CM proteins’ content

CMs obtained from the different culture conditions (CM_CTR_, CM_2D_ and CM_3D_) were assessed using the Human Angiogenesis Antibody Array (ab134000, Abcam, Cambridge, United Kingdom) (Fig. 1B), designed to identify 20 distinct molecules, according to the manufacturer's instructions and as previously reported [16,32]. This membrane array, already validated for ovine species [32], has the great advantage to detect molecules possessing a key role not only in angiogenesis but also in immunomodulation (i.e. IL-8, IL-6, INF-γ, MCP-1, ENA-78 and GRO) and in tenogenic and ECM remodeling processes (i.e. TGF-β1, IGF-1, PDGF-BB, RANTES, b-FGF, VEGF, VEGF-D, TIMP-1 and TIMP-2) (Supplementary 2). As previously reported, IL-6 was not detectable due to the highly species-specific antibody which impaired the detection of the relative sheep protein [16,32].

#### 2.4.2 AECs-derived CMs influence on in vitro angiogenesis

HUVECs (Lonza, Walkersville, MD, USA) were xeno-cultured for 24 h with the CM_CTR_, CM_2D_ and CM_3D_ (Fig. 1C), diluted in Endothelial Growth Medium-2 (EGM-2; 1:1) according to previously described protocols [32,33]. HUVECs cultured either with EGM-2 or EGM-2:α-MEM (1:1) were used as controls. HUVECs proliferation was assessed using crystal violet (1%) staining according to previously reported articles [32,33].

HUVECs tubule formation assay was performed at 24 h by using the *in vitro* Angiogenesis Assay Kit (Chemicon, Millipore, Sigma-Aldrich, Milan, Italy), following the manufacturer's instructions, and according to previous reports [[32], [33], [34]]. The images were acquired using an inverted phase-contrast microscope (Nikon) and the branching index (number of junctions formed per vessel area) was quantified with Image J using the angiogenesis analysis software (ImageJ 1.53 k, NIH, Bethesda, MD, USA).

#### 2.4.3 CM's immunomodulatory effect on PBMCs proliferation and Jurkat reporter cells activation

The immunomodulatory potential of CM_CTR_, CM_2D_ and CM_3D_ was evaluated using two different immune cell typologies: PBMCs and NF-κBeGFP reporter Jurkat JE6-1 T cell line (Jurkat reporter cells) (Fig. 1D).

The PBMCs proliferation test was performed using an autologous setting. To this aim, PBMCs were isolated by density gradient centrifugation with Ficoll-Paque PLUS (Cytiva, Milan, Italy) of 25 mL peripheral blood following manufacturer's instructions. PBMCs, at the concentration of 3 × 10^5^ cells, were then activated with 10 μg/mL of phytohemagglutinin (PHA; Sigma-Aldrich, Milan, Italy) and cultured for 72 h with CMs derived from all conditions. The morphology of PBMCs cultured in CMs were evaluated by inverted phase-contrast microscope (Nikon). Moreover, PBMCs proliferation was assessed by using CellTiter96 Aqueous One Solution Cell Proliferation Assay, according to the manufacturer's instructions (Promega, Milan, Italy). The absorbance (Abs) was measured at 490 nm by using EnSpire® Multimode Plate Reader (PerkinElmer, Waltham, MA, USA). The percentage of cell proliferation was estimated according to the following equation:

$$\%\;\text{PBMCs}\;\text{Proliferation}=\frac{\text{Abs}\;\text{of}\;\text{PBMCs}+\text{PHA}+\text{CM}}{\;\text{Abs}\;\text{of}\;\text{PBMCs}+\text{PHA}}\times 100$$

Jurkat reporter cells, a highly sensitive reporter cell line allowing fluorescence-based readout of NF-κB transcriptional activity [35,36], were kindly provided by Prof. Johannes Stöckl (Medical University of Wien). NF-κB signaling pathway activation through tool-like receptor (TLR4) stimulation was evaluated by exposing confluent Jurkat reporter cells [37] to LPS (100 ng/mL LPS-B5 ultrapure: InvivoGen) with CMs (1 mL) for 48 h at 37 °C in a humidiﬁed atmosphere with 5% CO_2_. Flow cytometry analysis was conducted to assess NF-κB activation through eGFP quantification, and the obtained results were reported as mean ± S.D. of the geometric mean of fluorescence intensity (gMFI) calculated for all viable reporter cells in the population.

### 2.5 Teno-inductive effect of engineered constructs on co-cultured AECs

The teno-inductive paracrine effect of AECs’ engineered constructs was assessed with long-term co-cultures with freshly isolated AECs (P0) (up to 14 days) conducted by using a *trans*-well system modifying a previously validated method [18,22].

**Fig. 2:**
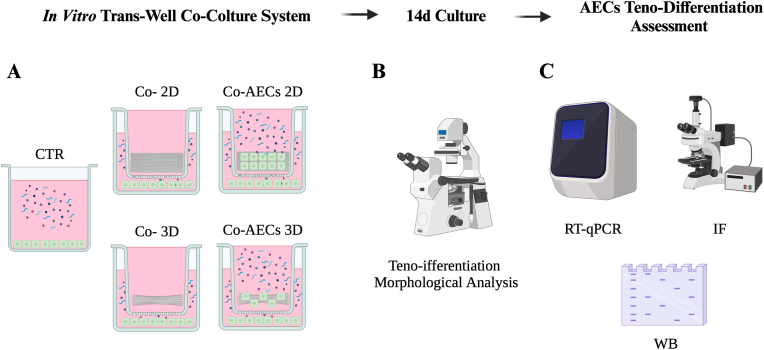
Experimental design for the analysis of the teno-inductive effect of engineered constructs on co-cultured AECs: (A) P0 AECs cultured on Petri dishes (CTR), co-culture between P0 AECs and non-engineered constructs (Co- 2D and Co- 3D) abbreviated within the text as CTR_INT_, and co-culture between P0 AECs and engineered constructs (Co-AECs 2D and Co-AECs 3D) for 14 d; (B) Teno-differentiation morphological analysis of co-cultured AECs; (C) Tenogenic differentiation assessment by evaluating SCX, THBS4, COL1 and TNMD gene regulation through real-time polymerase chain reaction (RT-qPCR), COL1 and TNMD protein expression by immunofluorescence (IF) and TNMD via Western Blot (WB).

P0 AECs were plated at 3 × 10^3^ cells/well and co-cultured through a *trans*-well chamber system (pore size 0.4 μm; Costar, NY, USA), with either AECs-2D (Co-AECs 2D) or AECs-3D (Co-AECs 3D). Moreover, P0 AECs, cultured at the same concentration as above, were co-cultured with either 2D and 3D constructs without being engineered with cells (Co- 2D and Co- 3D, respectively), and used as internal controls of the system (CTR_INT_). P0 AECs cultured on Petri dishes were used as CTR (Fig. 2A).

The teno-inductive microenvironment, generated by the differentiated engineered cells on both PLGA constructs (Supplementary 3), carried out as previously described [16], was tested by analyzing the tenogenic differentiation of co-cultured freshly isolated AECs by assessing the development of tendon-like structures and by analyzing the expression of tendon-related markers: *SCX*, *THBS4*, *COL1* and *TNMD* mRNAs and COL1 and TNMD proteins [18,22,38,39] (Fig. 2).

#### 2.5.1 Morphological and molecular assessment of co-cultured AECs teno-differentiation

The morphological changes within co-cultured AECs (Co-AECs 2D and Co-AECs 3D) and CTR groups (CTR and CTR_INT_) were observed during 14 days (14 d) of culture (Fig. 2B) as previously described [18,22]. Furthermore, samples were investigated, after 14 d of culture for *SCX*, *THBS4*, *COL1* and *TNMD* gene expression through real-time polymerase chain reaction (RT-qPCR), COL1 and TNMD protein expression by immunofluorescence (IF) [[18], [19], [20], [21]], and TNMD via Western Blot (WB) [18] (Fig. 2C).

In detail, for RT-qPCR, total RNA was extracted from AECs in the control groups (CTR and CTR_INT_), engineered on both constructs, and those subjected to co-culture using Total RNA Puriﬁcation Kit (Norgen Biotek Corp., Thorold, ON, Canada) according to the manufacturer's instructions, and to previously published work [16]. cDNA synthesis was performed starting from 1 μg of total RNA from each sample and following reverse transcriptase reaction with Random Hexamer Primers and Tetro Reverse Transcriptase (Bioline, Germany). RT-qPCR was performed via the SensiFAST TM SYBR Lo-ROX Kit (Bioline, Germany) using the primer sequences for the interested genes (Table 1). The reaction was executed via a 7500 Fast Real-Time PCR System (Life Technologies, Waltham, MA, USA), followed by melt proﬁle analysis (7500 Software version 2.3, Life Technologies, Waltham, MA, USA). Data was normalized on GAPDH endogenous reference gene expression and analyzed by using the comparative Ct (ΔΔCt) method to assess the relative gene expression ratio (2^−ΔΔCt^).

**Table 1:** Details on primer sequences.

Gene	Forward Primer **(**5′–3′**)**	Reverse Primer **(**5′–3′**)**	Product size (bp)
***GAPDH***	CCTGCACCACCAACTGCTTG	TTGAGCTCAGGGATGACCTTG	224
***SCX***	AACAGCGTGAACACGGCTTTC	TTTCTCTGGTTGCTGAGGCAG	299
***THBS4***	CCGCAGGTCTTTGACCTTCT	CAGGTAACGGAGGATGGCTTT	231
***COL1***	CGTGATCTGCGACGAACTTAA	GTCCAGGAAGTCCAGGTTGT	212
***TNMD***	TGGTGAAGACCTTCACTTTCC	TTAAACCCTCCCCAGCATGC	352

Primers used in Citeroni et al. [18] and Russo et al. [16].

Furthermore, for IF, all the sample were fixed for 10 min in 4% paraformaldehyde/PBS. After 3 washes with PBS, the samples were permeabilized in 0.05% Tween 20/1% BSA/PBS (10 min at RT) or 0.1% Triton X-100/PBS (5 min at RT) before COL1 and TNMD immunostainings, respectively. Blocking of non-specific bindings was performed by incubating cells at RT in PBS/1% BSA for 1 h. Primary antibodies for COL1 (EMD Millipore Corporation, Temecula, USA) and TNMD (ab203676, Abcam, Cambridge, United Kingdom) were diluted in PBS (1:100) and in 1% BSA/PBS (1:100), respectively. The samples were incubated overnight at 4 °C and subsequently treated with secondary antibodies: anti-Mouse Cy3 (Sigma-Aldrich, St. Louis, MO, USA) diluted in PBS (1:500) to reveal COL1 and anti-rabbit Alexa Fluor 488 (Molecular Probes, Göteborg, Sweden) diluted in PBS/BSA 1% (1:500) to evidence TNMD, for 1 h at room temperature (RT). Nuclear counterstaining was obtained with DAPI diluted in PBS (1:2000; Vector Laboratories, Milan, Italy) for 15 min at RT. Negative control of the reaction was performed by omitting primary antibodies. All controls performed were negative. Moreover, for WB analysis, the same TNMD primary antibody, as above, was diluted in Every Blot Blocking Buffer (1:500; Bio-Rad Laboratories, Milan, Italy). Anti-rabbit secondary antibody conjugated to horseradish peroxidase (HRP) (Santa Cruz, sc-2357, Heidelberg, Germany) diluted in TBS (1:2000), was used for antigen retrieval. Densitometric analysis was performed using Image J software (ImageJ 1.53 k, NIH, Bethesda, MD, USA). For each sample, TNMD protein was normalized to the corresponding housekeeping Tubulin expression (3873, Cell Signaling Technology, Danvers, Massachusetts, USA).

### 2.6 Statistical analysis

All experiments were conducted with three biological replicates for both AECs and PBMCs samples to evaluate inter-experimental variability. Each experiment was conducted in triplicate to assess intra-experimental variability. Data reported as the mean ± S.D. D'Agostino and Pearson tests were used to determine whether the results had a normal distribution. One-Way ANOVA and two tailored *t*-test were used to compare normally distributed data followed by Tukey post hoc analyses (GraphPad Prism 9, San Diego, CA, USA). Statistical significance was established at a *p*-value of at least 0.05.

## 3 Results

### 3.1 3D PLGA scaffold boosts the paracrine release of engineered AECs

**Fig. 3:**
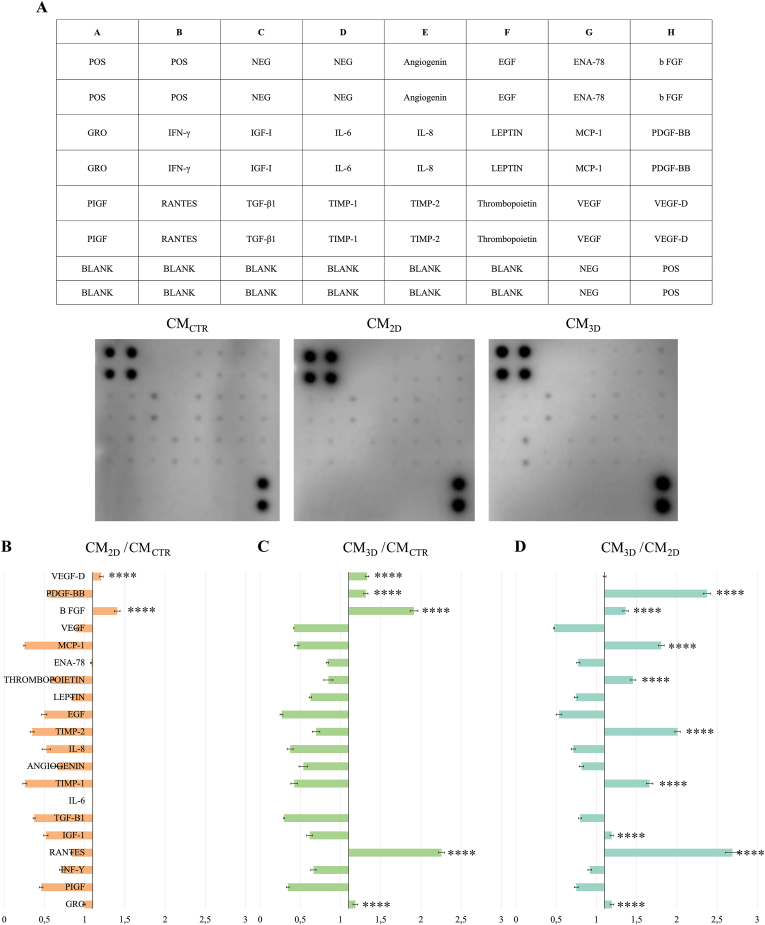
Characterization of AECs paracrine profile content. (A) Layout of the array and representative images of chemokines detection of all analyzed CMs (CM_CTR_, CM_2D_, and CM_3D_). Expression profile ratio obtained after proteins quantification by densitometric analysis of (B) CM_2D_/CM_CTR_, (C) CM_3D_/CM_CTR_ and (D) CM_3D_/CM_2D_. The different tested CMs were obtained from three biological replicates, conducted each in triplicate. **** Statistically significant values between the different studied groups (p < 0.0001)*.*

The CM analyses were performed by using a human cytokine antibody array made for the simultaneous detection of 20 factors (Fig. 3A and detailed in Supplementary 2). The protein profile confirmed a basal release of AECs (CM_CTR_) showing high levels of chemokines and angiogenic factors (Fig. 3A) according to a previous report [34]. However, the CM_2D_ and CM_3D_ (CM derived from AECs engineered either on PLGA fleeces or 3D scaffolds, respectively) showed a differential molecular release (Fig. 3A). In detail, the analyses of the molecules content ratio showed that, while the CM_2D_ had high levels of VEGF-D and b-FGF (*p* < 0.0001 *vs.* CM_CTR_; Fig. 3B), the CM_3D_, instead, showed an increased release of VEGF-D, PDGF-BB, b-FGF, RANTES and GRO proteins (*p* < 0.0001 *vs.* CM_CTR_; Fig. 3C). Interestingly, the highest levels of PDGF-BB, b-FGF, MCP-1, thrombopoietin, TIMP-2, TIMP-1, IGF-1, RANTES, and GRO proteins were detected within AEC-engineered 3D scaffold with respect to fleeces (*p* < 0.0001 CM_3D_ *vs*. CM_2D_; Fig. 3D).

### 3.2 CM derived from AECs’ engineered constructs are able to in vitro modulate angiogenesis

The angiogenic effect of the CM derived from all experimental conditions was biologically tested on HUVECs proliferation and tubule formation.

**Fig. 4:**
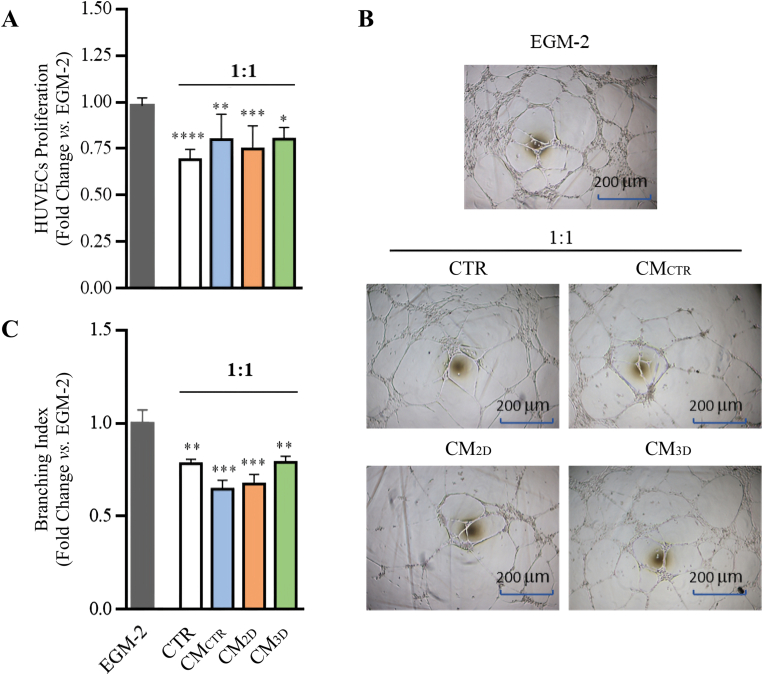
HUVEC proliferation and tubule formation analysis. (A) Representative histogram of HUVECs proliferation cultured either in CTR medium or AECs derived CM (CM_CTR_, CM_2D_, and CM_3D_) expressed as fold changes vs. HUVECs cultured in standard condition (EGM-2). (B) Representative images of tubule formation under different experimental conditions. (C) Representative histogram of branching index values, expressed as fold changes vs. EGM-2. The different tested CMs were obtained from three biological replicates and assessed for their angiogenic effect on three different HUVECs replicates. The experiment was conducted in triplicate. *, **, *** and **** expressed statistically significant values vs. EGM-2 for p < 0.05, p < 0.01, p < 0.001 and p < 0.0001, respectively.

The culture condition for HUVECs exposure to CMs was initially set up by studying the effect of dilution of EGM-2 (standard HUVECs growth medium) with α-MEM (AECs growth medium) [32]. The EGM-2:α-MEM dilution selected for the angiogenetic assays was 1:1 (CTR in Fig. 4A) since it allowed to maintain HUVECs proliferation on a value of approximately 70% [32] and significantly lower than EGM-2 (*p* < 0.001). All tested CMs were unable to influence HUVECs’ proliferation showing a comparable effect with respect to CTR medium (*p* > 0.05; Fig. 4A).

At the same time, all CMs showed a similar tubule formation as observed in HUVECs’ cultured in CTR medium (Fig. 4B). These results were confirmed by measuring the branching index, which resulted similar to those recorded within CTR medium (*p* > 0.005 *vs.* all CM groups; Fig. 4C). In particular, HUVECs showed a similar response among the different AECs culture conditions, developing tubule-like structures with an analogous degree of maturity and a comparable branch number and thickness (Fig. 4B and C).

### 3.3 3D scaffolds boost AECs paracrine immunosuppressive activity

Based on the differential immunomodulatory factors released within the different tested AECs-derived CM and detected by the membrane array (Fig. 3C), their biological immune potential was further investigated on PBMCs and on Jurkat reporter cells, designed for monitoring the NF-κB signal transduction pathway *in vitro*.

**Fig. 5:**
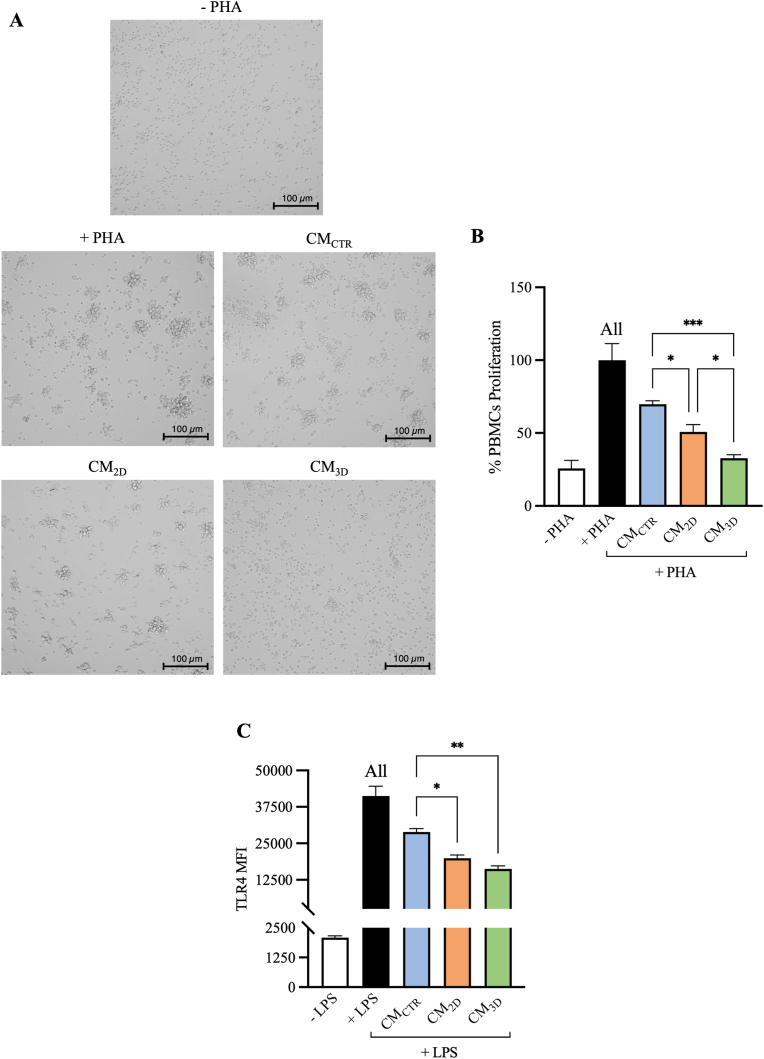
Immunosuppressive effect of AECs derived CM (CM_CTR_, CM_2D_, and CM_3D_) on PBMCs and Jurkat reporter cells. (A) Representative microscopical observations of basal (-PHA) and activated (+PHA) PBMCs exposed to different CMs. (B) PBMCs proliferation data was presented as histograms expressed as mean ± S.D. (C) Flow cytometry analysis of TLR4 activation on basal (-LPS) and activated (+LPS) in Jurkat reporter cells exposed to different CMs. TLR4 MFI data was presented as histograms expressed as mean ± S.D. The different tested CMs were obtained from three biological replicates and assessed for their immunomodulatory effect on three different PBMCs and Jurkat replicates. Each experiment was conducted in triplicate. All, *, ** and *** statistically significant values between the different studied groups (p < 0.01, p < 0.05, p < 0.01, and p < 0.001, respectively)*.*

CM collected from all AECs culture conditions suppressed significantly PHA-stimulated PBMCs proliferation (*p* < 0.05 *vs.* PHA; Fig. 5A and B). In particular, CM_CTR_ showed a basal inhibitory activity on PHA-induced PBMCs proliferation of about 30% (*p* < 0.05 *vs.* PHA; Fig. 5B). Interestingly, CM_2D_ and CM_3D_ resulted in damping further PHA-stimulated PBMCs proliferation, reaching inhibitory effects of about 49% and 67%, respectively (*p* < 0.05 and *p* < 0.001 *vs.* CM_CTR_; Fig. 5B). Of note, CM_3D_ showed the greatest immunosuppressive activity on PBMCs (*p* < 0.05 vs CM_2D_; Fig. 5B).

Additionally, all analyzed CM exerted an immunosuppressive role (Fig. 5C) by specifically influencing the LPS/TLR4 downstream activation of NF-κB signal transduction pathway visualized on Jurkat reporter cells (*p* < 0.01 *vs.* LPS; Fig. 5C). Interestingly, both CM_2D_ and CM_3D_ improved AECs’ paracrine immunosuppressive ability by significantly blunting TLR4 activation (CM_3D_ and CM_2D_ *vs.* CTR, *p* < 0.01 and *p* < 0.05, respectively; Fig. 5C).

### 3.4 AECs teno-differentiation is boosted when co-cultured with engineered 3D scaffolds

The teno-inductive paracrine activity of AECs-2D and AECs-3D were demonstrated by co-culturing them with freshly isolated AECs up to 14 days.

**Fig. 6:**
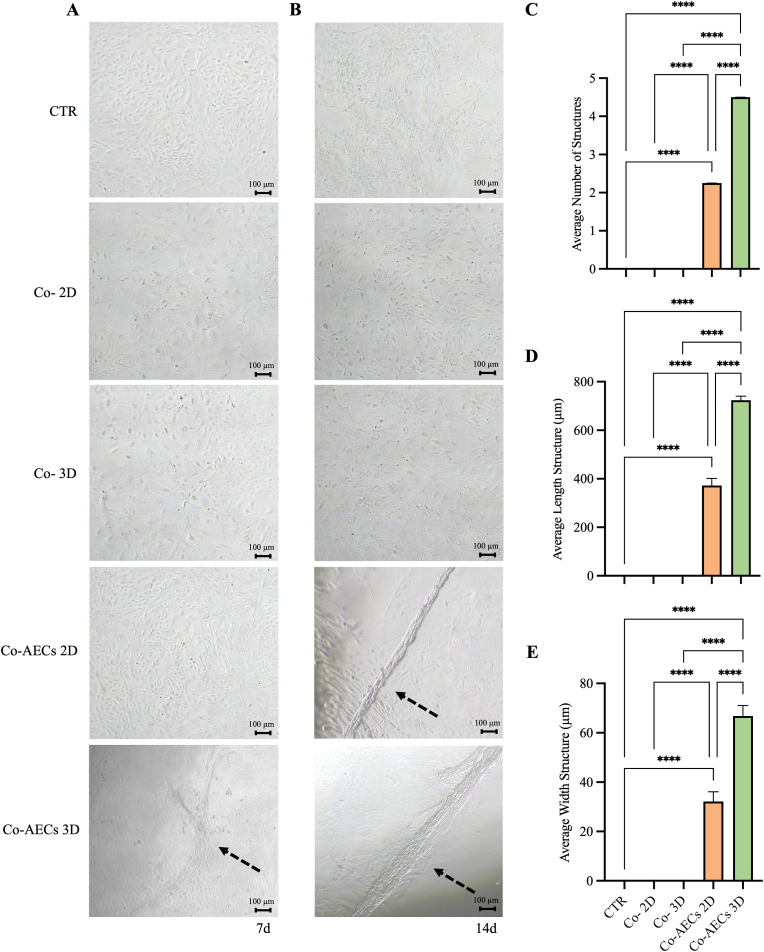
AECs engineered on PLGA constructs (AECs-2D and AECs-3D) paracrine teno-differentiative effect on co-cultured freshly isolated (P0) AECs (Co-AECs 2D and Co-AECs 3D, respectively). Morphological assessment of co-cultured AECs at (A) 7 d and (B) 14 d of culture. Histograms showing (C) the average number, (D) length, and (E) width of the formed tendon-like structures at 14 days culture within CTR, CTR_INT_ (Co- 2D and Co- 3D), Co-AECs 2D and Co-AECs 3D. The tendon-like structures are indicated with dotted black arrows. Data was presented as mean ± S.D. The co-cultured system was conducted in triplicate employing three AECs biological replicates. **** Statistically significant values between the different studied groups (p < 0.0001).

AECs exposed to the micro-environment conditioned from AECs-2D after 7 d of culture did not show any morphological change by preserving their monolayer organization similar to that recorded in AECs cultured under control condition (CTR) (Fig. 6A). Differently, AECs exposed to AECs-3D started to generate clustering and elongated structures (black arrow; Fig. 6A). After 14 d of co-culture, the tendon paracrine inductive effect of engineered constructs became more evident. Indeed, the co-cultured AECs exposed to both AECs-2D and AECs-3D developed elongated tendon-like structures (black arrow; Fig. 6B), while the CTR AECs and CTR_INT_ AECs maintained their monolayer organization by retaining their native cobblestone morphology (Fig. 6B).

The induction of tendon-like structures in AECs co-cultured with both engineered constructs were further quantified in terms of number, length, and width. Of note, engineered 3D scaffolds induced a signiﬁcantly higher number of tendon-like structures (4.5 *vs.* 2.25 for Co-AECs 3D *vs.* Co-AECs 2D, *p* < 0.0001; Fig. 6C) with a higher length and width (724 *vs.* 372 μm and 67 *vs.* 32 μm, respectively, Co-AECs 3D *vs.* Co-AECs 2D, *p* < 0.0001; Fig. 6D and E).

Both PLGA constructs confirmed to be teno-inductive for engineered AECs, according to previous data [16,[19], [20], [21]], which acquired an elongated tenocyte-like morphology, upregulated tendon related genes (*SCX*, *THBS4*, *COL1*, and *TNMD*) respect to CTR (at least *p* < 0.05), and expressed COL1 and TNMD protein (Supplementary 3).

**Fig. 7:**
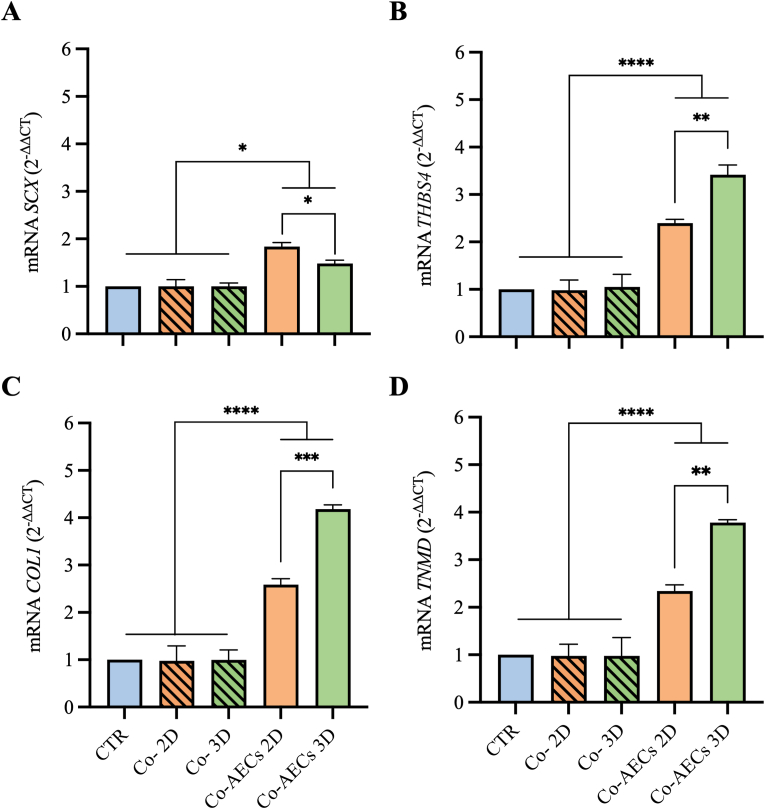
Tendon-specific gene expression profile in AECs co-cultured with engineered (Co-AECs 2D and Co-AECs 3D and non-engineered (Co-2D and Co-3D) PLGA fleeces and 3D scaffolds. RT-qPCR for (A) SCX, (B) THBS4, (C) COL1, and (D) TNMD gene expression after 14 d of culture. Data were normalized on CTR P0 AECs cultured on Petri dishes for 14 d and presented as mean ± S.D. The co-cultured system was conducted in triplicate employing three AECs biological replicates. *, **, ***, and **** Statistically significant values between the different studied groups (p < 0.05, p < 0.01, p < 0.001, and p < 0.0001, respectively).

Interestingly, AECs engineered on both PLGA constructs were able to condition the culture microenvironment, inducing the tenogenic differentiation of the co-cultured AECs (Fig. 7). Indeed, the formed tendon-like structures within co-cultured AECs expressed early (*SCX*) and late (*THBS4*, *COL1* and *TNMD*) tendon-related markers after 14 d of culture. In detail, the expression of all analyzed genes in both Co-AECs 2D and Co-AECs 3D was significantly higher compared to CTR and CTR_INT_ (*p* < 0.05; Fig. 7). Remarkably, all tendon related markers were significantly upregulated within Co-AECs 3D compared to Co-AECs 2D (*p* < 0.01; Fig. 7B, C, and D), except for *SCX* whose expression was higher in Co-AECs 2D (*p <* 0.05 *vs.* Co-AECs 3D; Fig. 7A).

**Fig. 8:**
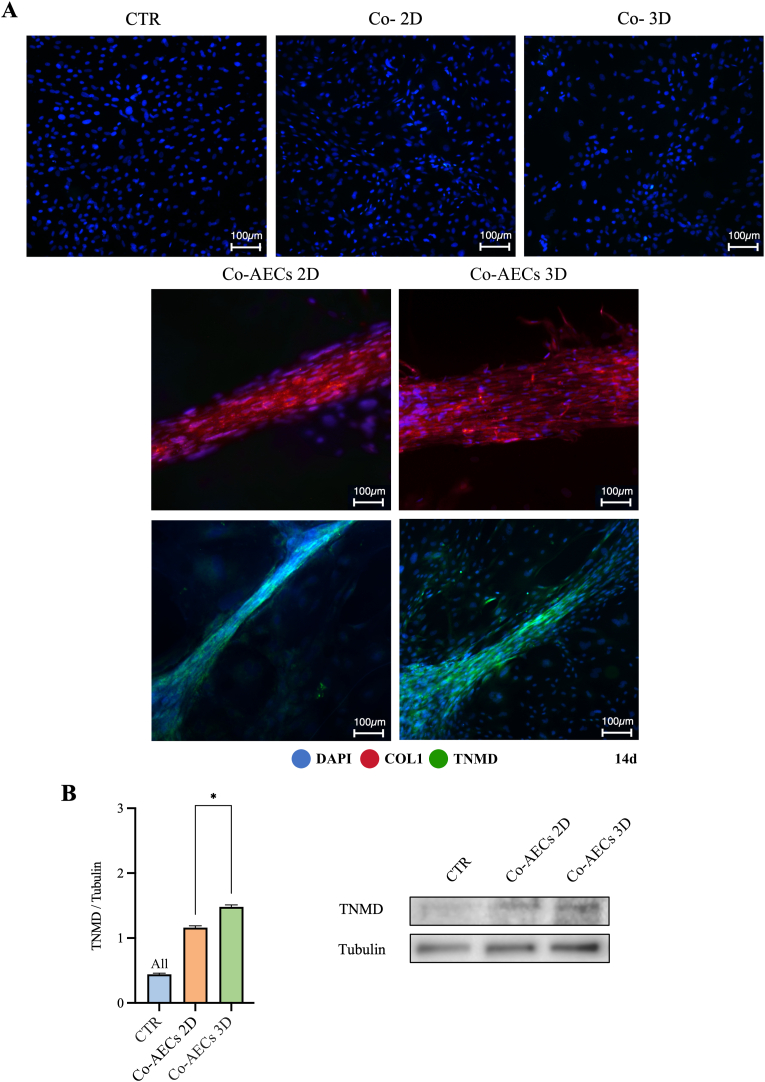
Assessment of tenogenic differentiation of AECs co-cultured with engineered constructs. (A) Representative IF images of CTR and CTR_INT_ (Co- 2D and Co- 3D), Co-AECs 2D and Co-AECs 3D, in which COL1 and TNMD are shown as red and green fluorescence, respectively. Cells' nuclei were counterstained with DAPI. (B) Representative WB analysis of TNMD expression within the samples. Data was presented as histograms expressed as mean ± S.D. The co-cultured system was conducted in triplicate employing three AECs biological replicates. All and * Statistically significant values between the different studied groups (p < 0.0001 and p < 0.05, respectively). (For interpretation of the references to color in this figure legend, the reader is referred to the Web version of this article.)

AECs’ tenogenic differentiation at the gene level was subsequently validated through protein analysis. It is worthy to highlight that CTR cells (CTR and CTR_INT_) retained a typical cuboidal morphology with round nuclei up to 14 days under standard culture, showing negativity for the analyzed tendon related markers: COL1 and TNMD (Fig. 8A). Instead, the cells contained in the newly formed tendon-like structures either Co-AECs 2D and Co-AECs 3D expressed both COL1 and TNMD proteins observed within their cytoplasm (Fig. 8A). Importantly, COL1 expression within these structures was not confined to the cytoplasm; the cells began depositing this protein extracellularly, forming a sort of ECM especially in Co-AECs 3D (Fig. 8A).

TNMD expression was quantified through WB analysis, revealing that Co-AECs 3D exhibited the highest expression compared to those co-cultured with AECs-2D (*p* < 0.05 *vs.* Co-AECs 2D; Fig. 8B).

## 4 Discussion

The comprehension of the intricate interplay between cells, materials, and their physical characteristics, such as topography and topology, is crucial to develop functional scaffolds that can effectively influence the regenerative process through the paracrine mechanisms involved in tissue regeneration [15,16,19,40].

This study demonstrates, for the first time, how the developed 3D scaffold, bio-mimicking the hierarchical tendon structure [16], was able to instruct and modulate AECs’ paracrine signaling by releasing molecules that modulate angiogenesis, immune system, and teno-differentiation, thus, establishing a crosstalk with the relevant target cells of these systems influencing their biological behavior. The paracrine and biological effect of the released molecules were influenced by the three-dimensionality (3D) of the PLGA electrospun tendon mimetic scaffold, showing a boosting effect on the cells with respect to fleeces (2D). These results strongly support the hypothesis that AECs engineered 3D scaffolds recreated a cell-to-cell organization able to better govern crucial regenerative processes that need to be orchestrated in order to promote a positive host response and tendon regeneration.

Both PLGA constructs promoted on engineered AECs a paracrine activity which exhibited a differential and potentiated release profile of bioactive molecules compared to that derived from naïve cells (CM_CTR_). It has been robustly demonstrated that AECs, *di per se*, secrete considerable amounts of angiogenic, anti-fibrotic, and anti-inflammatory factors, including VEGF, b-Fibroblast Growth Factor (b-FGF), angiogenin, and Insulin Growth Factor (IGF) [13,16,32], which represent all important bio-active factors able to exert a therapeutic function in different disease models [65]. However, the present results demonstrate that the interaction with an inductive substrate was able to modify the chemokines' secretion of the cells by inducing a positive releasing profile. In particular, engineering AECs within PLGA fleeces (2D) led to an increased release levels of VEGF-D and b-FGF factors. Cells’ paracrine VEGF-D and b-FGF secretion was further boosted through the interaction with 3D scaffolds together with an elevated release of RANTES and Platelet Derived Growth Factor-BB (PDGF-BB). Interestingly, all these bioactive molecules can likewise be considered key factors for angiogenesis and tendon healing. In particular, it has been recently demonstrated that VEGF-D, commonly related to blood and lymph vessel formation [41,42], elicits responses also in non-endothelial cells, such as macrophages and tenocytes, which express VEGF receptors, and stimulate cell proliferation in a dose-dependent manner [43]. Moreover, PDGF‐BB is crucial for increasing tenocyte proliferation and matrix remodeling, especially during the first phases of tendon healing [[44], [45], [46]], while b-FGF contributes to tendon repair by enhancing collagen synthesis [47]. RANTES has a role in promoting vascular and neuronal mediators essential for tendon development and healing process [70]. In contrast, Transforming Growth Factor-β (TGF-β), pivotal in tendon healing [[48], [49], [50], [51]], exhibited a decreased release within CM_2D_ and CM_3D_ with respect to CM_CTR_. However, TGF-β reduced secretion can be positively considered, since its modulation is necessary to avoid fibrotic scar tissue formation [52,53] and possibly support a correct regenerative process after scaffold transplantation.

These findings, together with our previous data that demonstrated the role of 3D scaffolds in enhancing AECs release of also other important immunomodulatory and tenogenic factors [16], confirm a positive boosting role of the 3D PLGA scaffolds in driving the pro-regenerative paracrine profile in engineered AECs. Of note, the present results demonstrate that the enhanced paracrine profile of AECs is effectively able to promote a pro-regenerative action by reproducing *in vitro* the functional tests for angiogenesis, immune response and tenogenesis. In particular, the secreted molecules had a biological effect on relevant target cells allowing to hypothesize a possible influence in driving tendon healing towards a pro-regenerative event.

Interestingly, engineering AECs on both PLGA fleeces and 3D scaffolds downregulated VEGF-A secretion, and consequently influenced HUVECs biological behavior in a similar way with respect to CTR media. To this regard, it can be hypothesized that the fabricated tendon biomimetic 3D scaffolds would blunt the angiogenic process during the inflammatory phase of tendon healing and in turn would impede an aberrant angiogenic effect also in terms of FBR when transplanted *in vivo*. Furthermore, the obtained results highlight that CM_CTR_ possess a paracrine effect on HUVECs proliferation and tubule formation confirming their angiogenic modulatory ability both *in vitro* [32] and *in vivo* [12,13]. Indeed, AECs transplantation in a sheep Achilles tendon injury model was able to downregulate VEGF mRNA and protein expressions and inducing a reduction and remodeling of the blood vessels within the host tissue stimulating hence tendon regeneration [9,12,13] as also demonstrated in other tissue models as bone [54].

Importantly, CM derived from AECs-engineered constructs (CM_2D_ and CM_3D_) exerted an immunosuppressive effect on different subtypes of analyzed immune cells, PBMCs and Jurkat reporter cells. CM derived from 3D scaffolds exhibited a significant dumping effect on PBMCs proliferation and also a significant inhibitory effect on Jurkat reporter cells’ activation and the related transcription factor NF-κB. This latter assumes a significant function in the activation of T cells, which can influence the immune response and consequently the repair processes in tendons [[55], [56], [57]]. In fact, the dysregulations of T cell activity and chronic inflammation may play a role in the persistence and chronicity of tendon injuries [[55], [56], [57]]. Signals produced within T cells during their engagement with molecules and cells can strongly influence the T cell receptor (TCR), consequently, either boosting or dumping the function of NF-κB [37,58,59].

Additionally, the secreted molecules by AECs-engineered PLGA constructs (AECs-2D and AECs-3D) generated a teno-inductive microenvironment able to promote the teno-differentiation of co-cultured freshly isolated cells at genotypic and phenotypic levels. In fact, the co-cultured AECs after 14 days not only acquired an elongated tenocyte-like morphology and formed tendon-like structures with a high morphological organization, but significantly upregulated early (*SCX*) and late (*THBS4, COL1,* and *TNMD*) tendon-related genes [16,19,38,39], and switched on COL1 and TNMD protein expression. This effect was significantly boosted when the cells were co-cultured with AECs-3D, highlighting the role of scaffold's topography and topology in tailoring a favorable teno-inductive microenvironment [16]. Indeed, the expression of *SCX* at 14 d of culture was higher in Co-AECs 2D respect to Co-AECs 3D, which instead expressed higher levels of the *SCX* downstream effector *TNMD* and the other late tendon-related genes (*THBS4* and *COL1*), suggesting an earlier and more efficient activation of SCX under 3D condition. This is indicative of a more sustained commitment of Co-AECs 3D towards the tenogenic lineage [19,60]. As a confirmation, TNMD protein was significantly expressed at higher levels in Co-AECs 3D respect to Co-AECs 2D. Of note, TNMD is considered a pivotal late marker of mature tenocytes *in vitro* and *in vivo,* as it orchestrates the formation of collagen fibrils [[60], [61], [62], [63]]. Moreover, THBS4 contributes to the regulation of ECM deposition and in the repair of myotendinous junction (MTJs) [64,65]. This can be correlated to the fact that, after 14 days of culture, both teno-differentiated AECs on engineered 3D scaffolds and within the tendon-like structures not only expressed COL1 in their cytoplasm but also within the scaffold and the extracellular space of the tendon-like structures, forming a sort of ECM. These results are in agreement with previous data obtained by the co-culture of AECs with fetal tendon explants [18,22]. Thus, this evidence strongly suggests that the engineered 3D scaffolds simulated a biologically active tendon structure which was able to specifically instruct an epithelial source of stem cells, that does not express any tendon-related protein, to respond to the released soluble factors into the CM and trigger their teno-differentiation by establishing an active *in vitro* dialogue. This study highlights the pivotal role of 3D construct topology in finely tuning AECs' paracrine function for a tailored tendon-like microenvironment. It has been previously demonstrated that scaffold topography and topology boost AECs' teno-differentiation and immunomodulation accompanied by a concurrent activation of the YAP mechanotransducer that translocated into the nuclei of the engineered cells [16]. Future research should explore molecular interactions and signaling cascades between YAP and AECs in order to offer insights for targeted strategies to optimize AECs' regenerative potential in tendon tissue engineering.

Thus, the 3D scaffold has a dual value: supporting the engineered stem cells to exert a tissue-specific *trans*-differentiating role and, at the same time, enhancing, through a paracrine action, the progenitor cells of the district to be regenerated.

These results are consistent with the concept of biomaterial scaffolds serving as physical and biochemical cues for guiding cell behavior and tissue regeneration. The tenogenic environment was preferably enhanced within the 3D scaffolds confirming the importance of the produced construct's topology and topography, mimic the hierarchical structure of a tendon in terms of fiber alignment and fiber size (1.27 μm) and tendon unit diameter (500 μm) [15,16,[19], [20], [21],^66^] in establishing a tailored microenvironment, which may have facilitated cell-cell interactions providing a supportive niche for stem cells, as AECs. Moreover, the mechanical properties of the fabricated 3D scaffolds closely resemble those of human patellar, rotator, and Achilles tendons, specifically in terms of ultimate tensile strength and Young's Modulus, according to Lomas et al. [5]. However, the fabricated scaffolds do not mimic the crimped fibers aspects, a key feature of tendon architecture, and tendon unit morphology. Future studies should explore scaffolds with enhanced biomimicry to better replicate natural tendon characteristics.

The instructive microenvironment of the engineered 3D tendon biomimetic scaffolds establishes a pro-regenerative crosstalk with the considered target cells favoring the interplay between blood vessels, immune system and tenogenic differentiation crucial for the regeneration of this tissue.

These proof of concepts of the pro-regenerative role of the 3D scaffolds collected *in vitro* are the essential prerequisite to move toward the confirmation of their tenogenic and pro-regenerative role by setting up *in vivo* in preclinical tendon injury models.

Overall, this study highlights the importance of the microenvironment and scaffold design in modulating cell behavior and functional outcomes [40], by stressing the strategic value of the *in vitro* models to predict also the complex dialogue between the engineered scaffolds and the somatic/immune/blood vessels districts, controlling, thus, the regeneration of the host diseased tissue. At the same time, the secreted molecules could be considered as a potential source of factors to be used in a cell-free therapy for tendon repair.

## Funding sources

This work was supported by:

- This project has received funding from the European Union's Horizon 2020 research and innovation programme under the Marie Skłodowska-Curie grant agreement No 955685 (www.p4fit.eu).
- The European Union – Next Generation EU. Project Code: ECS00000041; Project CUP: C43C22000380007; Project Title: Innovation, digitalization and sustainability for the diffused economy in Central Italy – VITALITY.

## CRediT authorship contribution statement

**Valentina Russo:** Writing – review & editing, Writing – original draft, Supervision, Resources, Project administration, Funding acquisition, Conceptualization. **Giuseppe Prencipe:** Writing – review & editing, Writing – original draft, Methodology, Investigation, Formal analysis, Data curation. **Annunziata Mauro:** Writing – review & editing, Writing – original draft. **Mohammad El Khatib:** Writing – review & editing, Writing – original draft, Methodology. **Arlette A. Haidar-Montes:** Formal analysis. **Nico Cambise:** Data curation. **Maura Turriani:** Methodology. **Johannes Stöckl:** Supervision, Resources. **Peter Steinberger:** Resources. **Loreto Lancia:** Methodology, Formal analysis. **Matthias Schnabelrauch:** Supervision. **Paolo Berardinelli:** Writing – review & editing. **Barbara Barboni:** Writing – review & editing, Resources, Funding acquisition, Conceptualization.

## Declaration of competing interest

The authors declare that they have no known competing financial interests or personal relationships that could have appeared to influence the work reported in this paper.

## Data Availability

Data will be made available on request.
