# Supplementary material for: Assessing the functional potential of conditioned media derived from amniotic epithelial stem cells engineered on 3D biomimetic scaffolds: An in vitro model for tendon regeneration
Source: Mater Today Bio. 2024 Feb 18;25:101001. doi: 10.1016/j.mtbio.2024.101001 (PMC10899023; doi:10.1016/j.mtbio.2024.101001)
Supplement: Multimedia component 1 [file mmc1.docx]

**Supplementary 1**

**
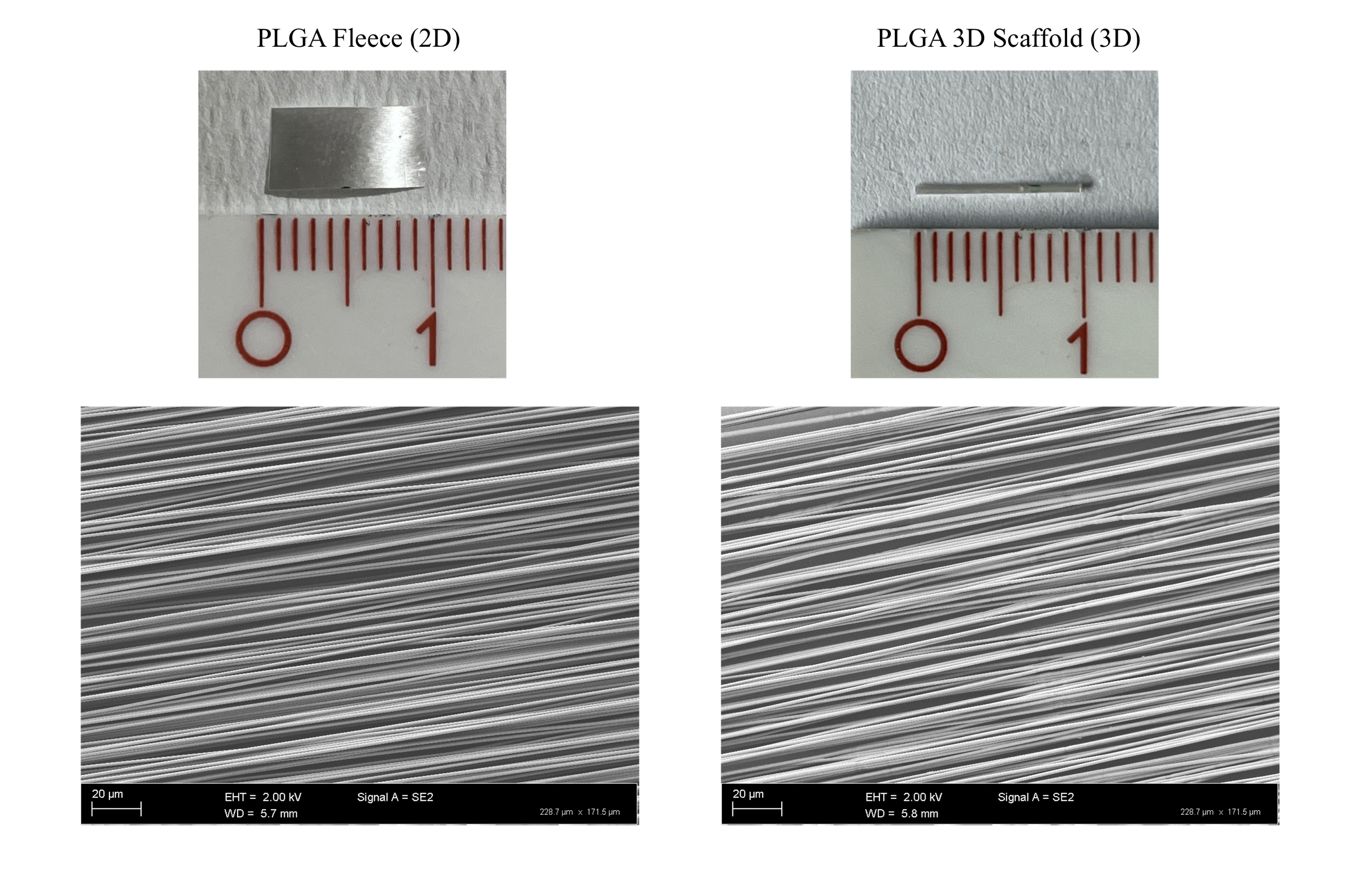
**Supplementary 1. Representative images of electrospun PLGA fleece (50 µm of thickness) and 3D scaffold (500 µm diameter) with the relative SEM micrographs showing the high alignment of the fibers. The complete characterization of the fabricated 3D scaffolds in terms of ultrastructure and mechanical properties has been published in Russo et al. [1]. In summary, the constructs’ fibers were of a diameter size of 1.27 ± 0.11 µm [1–3] mimicking the range of collagen fibers of native tissues (1 to 20 μm in diameter) and 3D scaffold with its diameter size mimicked tendon unit size [4]. The fabricated 3D scaffolds exhibit high mechanical properties: maximum load (8.38 ± 0.94 N), ultimate tensile strength (41.59 ± 4.27 MPa), elongation at break (115 ± 15%), and Young's Modulus (763.8 ± 151 MPa). The mechanical properties of the fabricated 3D scaffolds closely mimic those of human patellar, rotator, and Achilles tendons, specifically in terms of ultimate tensile strength and Young's Modulus, according to Lomas et al. [5].

**Supplementary 2**

Supplementary 2. Classification of molecules analyzed through the antibody array according to their other roles besides the angiogenic as immunomodulatory and tenogenic.

| Molecules | Angiogenic | Immunomodulatory | Tenogenic | References |
| --- | --- | --- | --- | --- |
| Growth-regulated oncogene (GRO) |  |  |  | [6,7] |
| Placental growth factor (PIGF) |  |  |  | [8,9] |
| Interferon-gamma (INF-γ) |  |  |  | [1,10–13] |
| Regulated on activation, normal T cell expressed and secreted (RANTES) |  |  |  | [1,14–17] |
| Insulin-like growth factor 1 (IGF-1) |  |  | * | [18–22] |
| Transforming growth factor β 1 (TGF-β1) |  |  | * | [1,19,23–25] |
| Interleukin 6 (IL-6) | n.d. | n.d. | n.d. | n.d. |
| Tissue inhibitor of metalloproteinases 1 (TIMP-1) |  |  |  | [26–28] |
| Angiogenin |  |  |  | [29] |
| Interleukin 8 (IL-8) |  |  |  | [1,21,30–33] |
| Tissue inhibitor of metalloproteinases 2 (TIMP-2) |  |  |  | [1,27,34–37] |
| Epidermal growth factor (EGF) |  |  |  | [38,39] |
| Leptin |  |  |  | [40,41] |
| Thrombopoietin |  |  |  | [42,43] |
| Epithelial- neutrophil activating peptide 78 (ENA-78) |  |  |  | [44] |
| Monocyte chemotactic protein 1 (MCP-1) |  |  |  | [1,14] |
| Vascular endothelial growth factor (VEGF) |  |  | * | [19,45–48] |
| Basic fibroblast growth factor (bFGF) |  |  | * | [19,22,25] |
| Platelet-derived growth factor BB (PDGF-BB) |  |  | * | [1,19,22,49–55] |
| Vascular endothelial growth factor (VEGF-D) |  |  | * | [22,56,57] |

Black (recognized role), red (pro-inflammatory role), green (anti-inflammatory role) and orange dots (pleiotropic role); n.d. not detected; * Involvement in teno-differentiation.

**Supplementary 3**


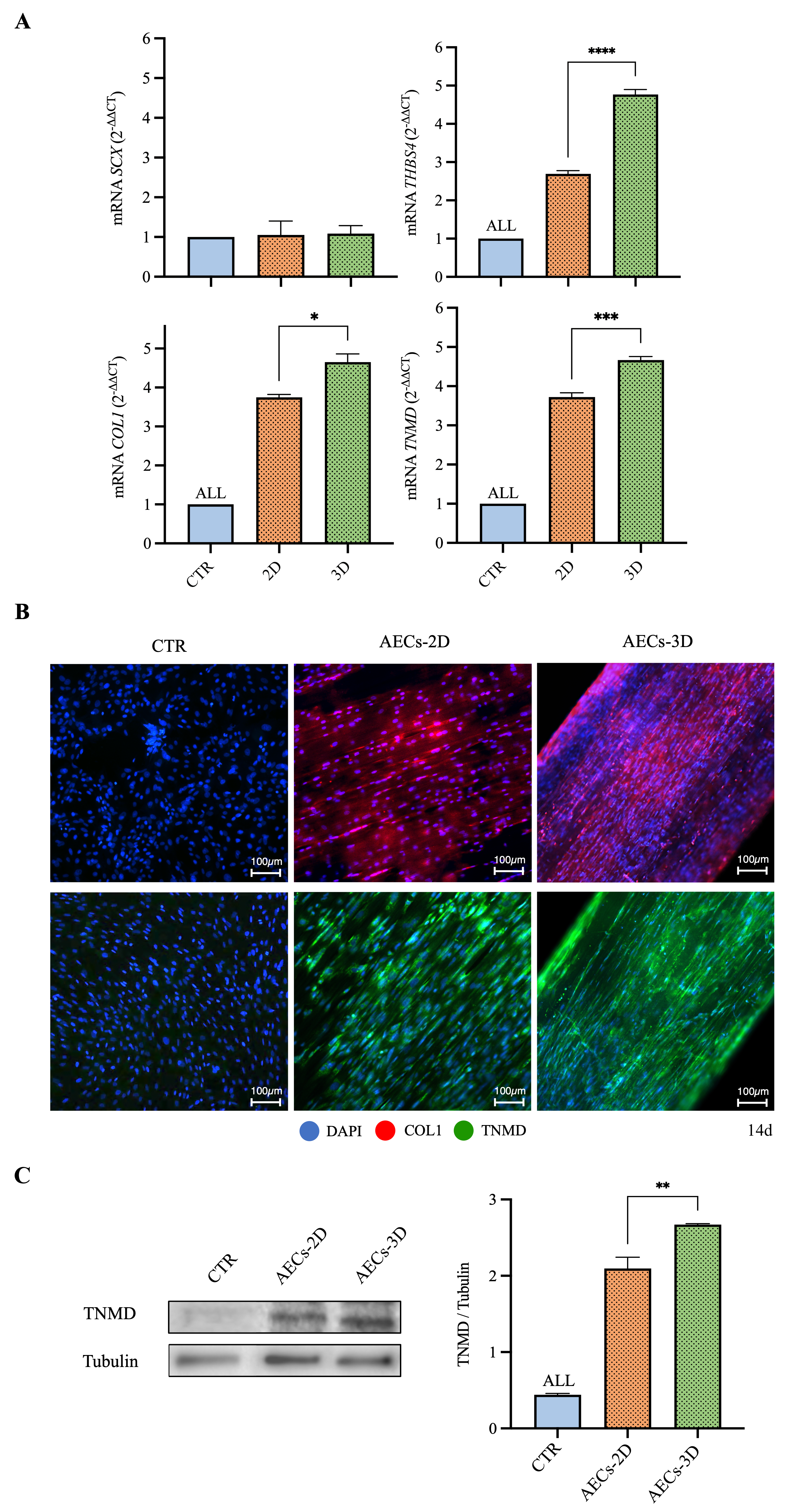


Supplementary 3. Assessment of teno-inductive potential of electrospun PLGA fleeces (2D) and 3D scaffolds (3D) on engineered AECs after 14 days of culture. (**A**) RT-qPCR of SCX, THBS4, COL1 and TNMD revealed an upregulation of all the late tendon-related markers (THSB4, COL1 and TNMD) in cells engineered on both 2D and 3D constructs. (**B**) Representative IF images in which COL1 and TNMD are shown in red and green fluorescence, respectively. It is evident the positivity to the analyzed proteins within the cytoplasm of the cells engineered on the constructs, with respect to CTR. Of note, COL1 in 3D was expressed also extracellularly within the scaffold. Cells’ nuclei were counterstained with DAPI. (**C**) WB analysis of TNMD expression within CTR, AECs-2D and AECs-3D, in which it is evident the highest TNMD expression in AECs-3D. The experiment was conducted in triplicate employing three AECs biological replicates. Data was presented as histograms expressed as mean ± S.D. All, *, **, ***, and **** Statistically significant values between the different studied groups (p<0.0001, p<0.05, p<0.01, p<0.001, and p<0.0001, respectively).

**Bibliography**

[1] V. Russo, M. El Khatib, G. Prencipe, A. Mauro, O. Di Giacinto, A.A. Haidar-Montes, F. Pulcini, B. Dufrusine, A. Cerveró-Varona, M. Faydaver, C. Di Berardino, E. Dainese, P. Berardinelli, M. Schnabelrauch, B. Barboni, Tendon 3D Scaffolds Establish a Tailored Microenvironment Instructing Paracrine Mediated Regenerative Amniotic Epithelial Stem Cells Potential, Biomedicines 10 (2022) 2578. https://doi.org/10.3390/biomedicines10102578.

[2] M. El Khatib, A. Mauro, M. Di Mattia, R. Wyrwa, M. Schweder, M. Ancora, F. Lazzaro, P. Berardinelli, L. Valbonetti, O. Di Giacinto, A. Polci, C. Cammà, M. Schnabelrauch, B. Barboni, V. Russo, Electrospun PLGA Fiber Diameter and Alignment of Tendon Biomimetic Fleece Potentiate Tenogenic Differentiation and Immunomodulatory Function of Amniotic Epithelial Stem Cells, Cells 9 (2020) 1–26. https://doi.org/10.3390/cells9051207.

[3] M. El Khatib, A. Mauro, R. Wyrwa, M. Di Mattia, M. Turriani, O. Di Giacinto, B. Kretzschmar, T. Seemann, L. Valbonetti, P. Berardinelli, M. Schnabelrauch, B. Barboni, V. Russo, Fabrication and Plasma Surface Activation of Aligned Electrospun PLGA Fiber Fleeces with Improved Adhesion and Infiltration of Amniotic Epithelial Stem Cells Maintaining their Teno-inductive Potential, Molecules 25 (2020) 3176. https://doi.org/10.3390/molecules25143176.

[4] C.G. Fuentes-Corona, J. Licea-Rodriguez, R. Younger, R. Rangel-Rojo, E.O. Potma, I. Rocha-Mendoza, Second harmonic generation signal from type I collagen fibers grown in vitro, Biomed. Opt. Express 10 (2019) 6449. https://doi.org/10.1364/BOE.10.006449.

[5] A.J. Lomas, C.N.M. Ryan, A. Sorushanova, N. Shologu, A.I. Sideri, V. Tsioli, G.C. Fthenakis, A. Tzora, I. Skoufos, L.R. Quinlan, G. O’Laighin, A.M. Mullen, J.L. Kelly, S. Kearns, M. Biggs, A. Pandit, D.I. Zeugolis, The past, present and future in scaffold-based tendon treatments, Advanced Drug Delivery Reviews 84 (2015) 257–277. https://doi.org/10.1016/j.addr.2014.11.022.

[6] M. Caunt, L. Hu, T. Tang, P.C. Brooks, S. Ibrahim, S. Karpatkin, Growth-regulated oncogene is pivotal in thrombin-induced angiogenesis, Cancer Res 66 (2006) 4125–4132. https://doi.org/10.1158/0008-5472.CAN-05-2570.

[7] S.K. Ahuja, P.M. Murphy, The CXC Chemokines Growth-regulated Oncogene (GRO) α, GROβ, GROγ, Neutrophil-activating Peptide-2, and Epithelial Cell-derived Neutrophil-activating Peptide-78 Are Potent Agonists for the Type B, but Not the Type A, Human Interleukin-8 Receptor*, Journal of Biological Chemistry 271 (1996) 20545–20550. https://doi.org/10.1074/jbc.271.34.20545.

[8] S. De Falco, The discovery of placenta growth factor and its biological activity, Exp Mol Med 44 (2012) 1–9. https://doi.org/10.3858/emm.2012.44.1.025.

[9] S. Sissaoui, S. Egginton, L. Ting, A. Ahmed, P.W. Hewett, Hyperglycaemia up-regulates placental growth factor (PlGF) expression and secretion in endothelial cells via suppression of PI3 kinase-Akt signalling and activation of FOXO1, Sci Rep 11 (2021) 16344. https://doi.org/10.1038/s41598-021-95511-8.

[10] G. Kak, M. Raza, B.K. Tiwari, Interferon-gamma (IFN-γ): Exploring its implications in infectious diseases, Biomolecular Concepts 9 (2018) 64–79. https://doi.org/10.1515/bmc-2018-0007.

[11] Z. Zha, F. Bucher, A. Nejatfard, T. Zheng, H. Zhang, K. Yea, R.A. Lerner, Interferon-γ is a master checkpoint regulator of cytokine-induced differentiation, Proc. Natl. Acad. Sci. U.S.A. 114 (2017). https://doi.org/10.1073/pnas.1706915114.

[12] L.K. Teixeira, B.P. Fonseca, B.A. Barboza, J.P. Viola, The role of interferon-γ on immune and allergic responses, (n.d.) 8.

[13] H.L. Henry, A.W. Norman, Encyclopedia of Hormones, n.d. http://www.sciencedirect.com:5070/referencework/9780123411037/encyclopedia-of-hormones (accessed December 13, 2023).

[14] A. Stålman, D. Bring, P.W. Ackermann, Chemokine expression of CCL2, CCL3, CCL5 and CXCL10 during early inflammatory tendon healing precedes nerve regeneration: an immunohistochemical study in the rat, Knee Surg Sports Traumatol Arthrosc 23 (2015) 2682–2689. https://doi.org/10.1007/s00167-014-3010-9.

[15] E.P. Adler, C.A. Lemken, N.S. Katchen, R.A. Kurt, A dual role for tumor-derived chemokine RANTES (CCL5), Immunology Letters 90 (2003) 187–194. https://doi.org/10.1016/j.imlet.2003.09.013.

[16] S.A. Agere, N. Akhtar, J.M. Watson, S. Ahmed, RANTES/CCL5 Induces Collagen Degradation by Activating MMP-1 and MMP-13 Expression in Human Rheumatoid Arthritis Synovial Fibroblasts, Front. Immunol. 8 (2017) 1341. https://doi.org/10.3389/fimmu.2017.01341.

[17] K. Kimura, M. Nagano, G. Salazar, T. Yamashita, I. Tsuboi, H. Mishima, S. Matsushita, F. Sato, K. Yamagata, O. Ohneda, The Role of CCL5 in the Ability of Adipose Tissue-Derived Mesenchymal Stem Cells to Support Repair of Ischemic Regions, Stem Cells and Development 23 (2014) 488–501. https://doi.org/10.1089/scd.2013.0307.

[18] I. Miescher, J. Rieber, M. Calcagni, J. Buschmann, In Vitro and In Vivo Effects of IGF-1 Delivery Strategies on Tendon Healing: A Review, International Journal of Molecular Sciences 24 (2023) 2370. https://doi.org/10.3390/ijms24032370.

[19] X. Liu, B. Zhu, Y. Li, X. Liu, S. Guo, C. Wang, S. Li, D. Wang, The Role of Vascular Endothelial Growth Factor in Tendon Healing, Front Physiol 12 (2021) 766080. https://doi.org/10.3389/fphys.2021.766080.

[20] A. Shojaee, A. Parham, Strategies of tenogenic differentiation of equine stem cells for tendon repair: current status and challenges, Stem Cell Research & Therapy 10 (2019) 181. https://doi.org/10.1186/s13287-019-1291-0.

[21] I.M. Ellis, L.V. Schnabel, A.K. Berglund, Defining the profile: Characterizing cytokines in tendon injury to improve clinical therapy, Journal of Immunology and Regenerative Medicine 16 (2022) 100059. https://doi.org/10.1016/j.regen.2022.100059.

[22] M. Lin, W. Li, X. Ni, Y. Sui, H. Li, X. Chen, Y. Lu, M. Jiang, C. Wang, Growth factors in the treatment of Achilles tendon injury, Front Bioeng Biotechnol 11 (2023) 1250533. https://doi.org/10.3389/fbioe.2023.1250533.

[23] G.-K. Tan, B.A. Pryce, A. Stabio, J.V. Brigande, C. Wang, Z. Xia, S.F. Tufa, D.R. Keene, R. Schweitzer, Tgfβ signaling is critical for maintenance of the tendon cell fate, eLife 9 (2020) e52695. https://doi.org/10.7554/eLife.52695.

[24] Y. Li, X. Liu, X. Liu, Y. Peng, B. Zhu, S. Guo, C. Wang, D. Wang, S. Li, Transforming growth factor-β signalling pathway in tendon healing, Growth Factors 40 (2022) 98–107. https://doi.org/10.1080/08977194.2022.2082294.

[25] Y. Liu, C.-W. Suen, J. Zhang, G. Li, Current concepts on tenogenic differentiation and clinical applications, Journal of Orthopaedic Translation 9 (2017) 28–42. https://doi.org/10.1016/j.jot.2017.02.005.

[26] Q.X.A. Sang, Complex role of matrix metalloproteinases in angiogenesis, Cell Res 8 (1998) 171–177. https://doi.org/10.1038/cr.1998.17.

[27] S. Minkwitz, A. Schmock, A. Kurtoglu, S. Tsitsilonis, S. Manegold, B. Wildemann, F. Klatte-Schulz, Time-Dependent Alterations of MMPs, TIMPs and Tendon Structure in Human Achilles Tendons after Acute Rupture, Int J Mol Sci 18 (2017) 2199. https://doi.org/10.3390/ijms18102199.

[28] N. Altmann, C. Bowlby, H. Coughlin, Z. Belacic, S. Sullivan, S. Durgam, Interleukin-6 upregulates extracellular matrix gene expression and transforming growth factor β1 activity of tendon progenitor cells, BMC Musculoskelet Disord 24 (2023) 907. https://doi.org/10.1186/s12891-023-07047-9.

[29] S.H. Lee, K.W. Kim, K.-M. Min, K.-W. Kim, S.-I. Chang, J.C. Kim, Angiogenin reduces immune inflammation via inhibition of TANK-binding kinase 1 expression in human corneal fibroblast cells, Mediators Inflamm 2014 (2014) 861435. https://doi.org/10.1155/2014/861435.

[30] P.W. Ackermann, E. Domeij-Arverud, P. Leclerc, P. Amoudrouz, G.A. Nader, Anti-inflammatory cytokine profile in early human tendon repair, Knee Surg Sports Traumatol Arthrosc 21 (2013) 1801–1806. https://doi.org/10.1007/s00167-012-2197-x.

[31] Y.M. Zhu, S.J. Webster, D. Flower, P.J. Woll, Interleukin-8/CXCL8 is a growth factor for human lung cancer cells, Br J Cancer 91 (2004) 1970–1976. https://doi.org/10.1038/sj.bjc.6602227.

[32] N. Mukaida, Pathophysiological roles of interleukin-8/CXCL8 in pulmonary diseases, American Journal of Physiology-Lung Cellular and Molecular Physiology 284 (2003) L566–L577. https://doi.org/10.1152/ajplung.00233.2002.

[33] D.J. Brat, A.C. Bellail, E.G. Van Meir, The role of interleukin-8 and its receptors in gliomagenesis and tumoralangiogenesis, Neuro-Oncology 7 (2005) 122–133. https://doi.org/10.1215/S1152851704001061.

[34] T. Koike, R.B. Vernon, M.D. Gooden, E. Sadoun, M.J. Reed, Inhibited Angiogenesis in Aging: A Role for TIMP-2, The Journals of Gerontology Series A: Biological Sciences and Medical Sciences 58 (2003) B798–B805. https://doi.org/10.1093/gerona/58.9.B798.

[35] J. Batra, A.S. Soares, C. Mehner, E.S. Radisky, Matrix Metalloproteinase-10/TIMP-2 Structure and Analyses Define Conserved Core Interactions and Diverse Exosite Interactions in MMP/TIMP Complexes, PLoS ONE 8 (2013) e75836. https://doi.org/10.1371/journal.pone.0075836.

[36] W. Wang, D. Li, L. Xiang, M. Lv, L. Tao, T. Ni, J. Deng, X. Gu, S. Masatara, Y. Liu, Y. Zhou, TIMP-2 inhibits metastasis and predicts prognosis of colorectal cancer via regulating MMP-9, Cell Adhesion & Migration 13 (2019) 272–283. https://doi.org/10.1080/19336918.2019.1639303.

[37] S.E. Hoegy, H.-R. Oh, M.L. Corcoran, W.G. Stetler-Stevenson, Tissue Inhibitor of Metalloproteinases-2 (TIMP-2) Suppresses TKR-Growth Factor Signaling Independent of Metalloproteinase Inhibition, Journal of Biological Chemistry 276 (2001) 3203–3214. https://doi.org/10.1074/jbc.M008157200.

[38] R.B. Vega, K. Matsuda, J. Oh, A.C. Barbosa, X. Yang, E. Meadows, J. McAnally, C. Pomajzl, J.M. Shelton, J.A. Richardson, G. Karsenty, E.N. Olson, Histone Deacetylase 4 Controls Chondrocyte Hypertrophy during Skeletogenesis, Cell 119 (2004) 555–566. https://doi.org/10.1016/j.cell.2004.10.024.

[39] L. Wang, Z. Huang, W. Huang, X. Chen, P. Shan, P. Zhong, Z. Khan, J. Wang, Q. Fang, G. Liang, Y. Wang, Inhibition of epidermal growth factor receptor attenuates atherosclerosis via decreasing inflammation and oxidative stress, Sci Rep 7 (2017) 45917. https://doi.org/10.1038/srep45917.

[40] V. Abella, M. Scotece, J. Conde, J. Pino, M.A. Gonzalez-Gay, J.J. Gómez-Reino, A. Mera, F. Lago, R. Gómez, O. Gualillo, Leptin in the interplay of inflammation, metabolism and immune system disorders, Nat Rev Rheumatol 13 (2017) 100–109. https://doi.org/10.1038/nrrheum.2016.209.

[41] K.H. Collins, W. Herzog, G.Z. MacDonald, R.A. Reimer, J.L. Rios, I.C. Smith, R.F. Zernicke, D.A. Hart, Obesity, Metabolic Syndrome, and Musculoskeletal Disease: Common Inflammatory Pathways Suggest a Central Role for Loss of Muscle Integrity, Frontiers in Physiology 9 (2018). https://www.frontiersin.org/articles/10.3389/fphys.2018.00112 (accessed December 14, 2023).

[42] J. Pasquet, B.S. Gross, M.-P. Gratacap, L. Quek, S. Pasquet, B. Payrastre, G. van Willigen, J.C. Mountford, S.P. Watson, Thrombopoietin potentiates collagen receptor signaling in platelets through a phosphatidylinositol 3-kinase–dependent pathway, Blood 95 (2000) 3429–3434. https://doi.org/10.1182/blood.V95.11.3429.

[43] W.-H. Xu, L.-C. Mo, M.-H. Shi, H. Rao, X.-Y. Zhan, M. Yang, Correlation between thrombopoietin and inflammatory factors, platelet indices, and thrombosis in patients with sepsis: A retrospective study, World J Clin Cases 10 (2022) 4072–4083. https://doi.org/10.12998/wjcc.v10.i13.4072.

[44] M.M. Halloran, J.M. Woods, R.M. Strieter, Z. Szekanecz, M.V. Volin, S. Hosaka, G.K. Haines III, S.L. Kunkel, M.D. Burdick, A. Walz, A.E. Koch, The Role of an Epithelial Neutrophil-Activating Peptide-78-Like Protein in Rat Adjuvant-Induced Arthritis1, The Journal of Immunology 162 (1999) 7492–7500. https://doi.org/10.4049/jimmunol.162.12.7492.

[45] Y. Hou, Z. Mao, X. Wei, L. Lin, L. Chen, H. Wang, X. Fu, J. Zhang, C. Yu, Effects of transforming growth factor-beta1 and vascular endothelial growth factor 165 gene transfer on Achilles tendon healing, Matrix Biol 28 (2009) 324–335. https://doi.org/10.1016/j.matbio.2009.04.007.

[46] B. Barboni, V. Russo, V. Curini, A. Mauro, A. Martelli, A. Muttini, N. Bernabò, L. Valbonetti, M. Marchisio, O. Di Giacinto, P. Berardinelli, M. Mattioli, Achilles tendon regeneration can be improved by amniotic epithelial cell allotransplantation, Cell Transplant 21 (2012) 2377–2395. https://doi.org/10.3727/096368912X638892.

[47] M.E.J. Reinders, M. Sho, A. Izawa, P. Wang, D. Mukhopadhyay, K.E. Koss, C.S. Geehan, A.D. Luster, M.H. Sayegh, D.M. Briscoe, Proinflammatory functions of vascular endothelial growth factor in alloimmunity, J Clin Invest 112 (2003) 1655–1665. https://doi.org/10.1172/JCI200317712.

[48] A. Kraus, D. Sattler, M. Wehland, R. Luetzenberg, N. Abuagela, M. Infanger, Vascular Endothelial Growth Factor Enhances Proliferation of Human Tenocytes and Promotes Tenogenic Gene Expression, Plast Reconstr Surg 142 (2018) 1240–1247. https://doi.org/10.1097/PRS.0000000000004920.

[49] S. Gehmert, S. Gehmert, M. Hidayat, M. Sultan, A. Berner, S. Klein, J. Zellner, M. Müller, L. Prantl, Angiogenesis: The role of PDGF-BB on Adiopse-tissue derived Stem Cells (ASCs), Clinical Hemorheology and Microcirculation 48 (2011) 5–13. https://doi.org/10.3233/CH-2011-1397.

[50] S. Thomopoulos, F.L. Harwood, M.J. Silva, D. Amiel, R.H. Gelberman, Effect of Several Growth Factors on Canine Flexor Tendon Fibroblast Proliferation and Collagen Synthesis In Vitro, The Journal of Hand Surgery 30 (2005) 441–447. https://doi.org/10.1016/j.jhsa.2004.12.006.

[51] S. Thomopoulos, M. Zaegel, R. Das, F.L. Harwood, M.J. Silva, D. Amiel, S. Sakiyama-Elbert, R.H. Gelberman, PDGF-BB released in tendon repair using a novel delivery system promotes cell proliferation and collagen remodeling, J. Orthop. Res. 25 (2007) 1358–1368. https://doi.org/10.1002/jor.20444.

[52] Z. Mihaylova, R. Tsikandelova, P. Sanimirov, N. Gateva, V. Mitev, N. Ishkitiev, Role of PDGF-BB in proliferation, differentiation and maintaining stem cell properties of PDL cells in vitro, Archives of Oral Biology 85 (2018) 1–9. https://doi.org/10.1016/j.archoralbio.2017.09.019.

[53] H. Lin, B. Chen, W. Sun, W. Zhao, Y. Zhao, J. Dai, The effect of collagen-targeting platelet-derived growth factor on cellularization and vascularization of collagen scaffolds, Biomaterials 27 (2006) 5708–5714. https://doi.org/10.1016/j.biomaterials.2006.07.023.

[54] M. Zhang, F. Jiang, X. Zhang, S. Wang, Y. Jin, W. Zhang, X. Jiang, The Effects of Platelet-Derived Growth Factor-BB on Human Dental Pulp Stem Cells Mediated Dentin-Pulp Complex Regeneration, Stem Cells Translational Medicine 6 (2017) 2126–2134. https://doi.org/10.1002/sctm.17-0033.

[55] Y. Chen, L. Jiang, K. Lyu, J. Lu, L. Long, X. Wang, T. Liu, S. Li, A Promising Candidate in Tendon Healing Events—PDGF-BB, Biomolecules 12 (2022) 1518. https://doi.org/10.3390/biom12101518.

[56] S.A. Stacker, M.G. Achen, Emerging Roles for VEGF-D in Human Disease, Biomolecules 8 (2018) 1. https://doi.org/10.3390/biom8010001.

[57] H. Tempfer, G. Spitzer, C. Lehner, A. Wagner, R. Gehwolf, J. Fierlbeck, N. Weissenbacher, M. Jessen, L.M. Heindl, A. Traweger, VEGF‐D‐mediated signaling in tendon cells is involved in degenerative processes, The FASEB Journal 36 (2022). https://doi.org/10.1096/fj.202100773RRR.
